# Medical Opinion and Movement

**Published:** 1911-05-20

**Authors:** 


### Medical Opinion and Movement.

### Eustachian Catheterism.

At a meeting of the *Académie de Médecine* Dr. Lermoyez discussed the use and abuse of the Eustachian catheter. He considers that it is employed much too indiscriminately. It is often useless, sometimes dangerous. In acute otitis media it increases pain, favours a spread of the infection, and may make it light up again when it is already receding. In chronic catarrhal otitis media it may be of some service, provided it is only used during the subsidence of a sub-acute attack, but it is especially to be avoided in chronic dry otitis media, and more particularly in the juvenile otosclerosis which often attacks young girls and leads to complete deafness. It may set up subjective noises or increase the intensity of those already existing, and it may accelerate the progress of deafness. Catheterism of the Eustachian tube should therefore be reserved only for catarrhal otitis media during convalescence, and even in such cases attention to the nose is of chief importance in the cure of the affection. In the question of the treatment of deafness there are far more contra-indications than indications for the use of the Eustachian catheter. Not 90 per cent. but rather 10 per cent. of those suffering from deafness are amenable to catheter treatment. Such in brief outline is the teaching of Dr. Lermoyez.

### Gastro-Intestinal Reflexes.

In the *Zeitschrift für Physiologischen Chemie* there appears a contribution by Drs. Best and Cohnheim on their research work to determine the nature of certain gastro-intestinal reflexes. Pavlov has already shown that the evacuation of the stomach is retarded by the introduction of acid or oil into the duodenum. Cohnheim and Baumstark have further shown that the same retarding effect is produced by introducing hydrochloric acid into the lowest portion of the small intestine. The present authors experimented with dogs. Three fistulæ were formed, one in the duodenum, one in the upper part of the jejunum, and one about the middle of the small intestine. When acid or oil was introduced into either of these fistulæ, no chyme passed through the pylorus into the intestine. If, however, the mucous membrane of the intestine was anæsthetised with novocaine, the injection of oil or acid was without effect, showing that the mechanism of the phenomenon was a nervous reflex depending upon centripetal nerves of the small intestine. Further experiments were carried out to study the normal movements of the pylorus, and it was found that not only the presence of hydrochloric acid in the stomach induces opening of the pylorus and sets up peristaltic movements of the pyloric end, but that a psychic factor also is involved. The psychic factor in gastric secretion has already been demonstrated by Pavlov and others, but Drs. Best and Cohnheim find that the sight of food and drink excites also the motility of the stomach in hungry and thirsty dogs. Further, if water be introduced by an œsophageal fistula the evacuation through the pylorus is much more rapid in a thirsty dog than in a dog not thirsty.

### An Abdominal Sign of Pericarditis.

The abdominal signs in the early stage of pleuro-pneumonia that may occasion a mistaken diagnosis of some abdominal affection are already well known. Dr. Wynter draws attention to the fact that similar abdominal signs may present themselves in cases of pericarditis and lead to a mistaken diagnosis. The effect upon the phrenic nerve is bilateral, instead of unilateral, as in the case of some affection of the lung or pleura, so that a complete absence of respiratory abdominal expansion results, as in cases of abdominal affections. Dr. Wynter has observed two cases in which this sign has led to the supposition of an abdominal lesion, the presence of a pericarditis only being demonstrated by subsequent developments. The first patient was a young woman who was suddenly seized with acute epigastric pains. Owing to the absence of all respiratory abdominal movement a perforated gastric ulcer was thought probable, and surgical intervention was considered, but on the fourth day a pericardial rub was detected accompanied by intense dyspnœa and pericardial pain, the abdominal wall remaining immobile. The second case was that of a man aged forty, admitted into hospital for abdominal pain, anorexia, vomiting, fever, and pulse 92. The patient lay on his back with the legs drawn up. The abdominal muscles on the right side were tense, and there was tenderness in the right iliac fossa. The respiratory abdominal movements were hardly perceptible. There was a doubling of the second heart sound. Appendicitis was diagnosed. The condition subsided. Five weeks later laparotomy was performed for the removal of the offending appendix, which was, however, found to be quite normal. Five days later a second attack occurred similar to the first, but this time there was a well-marked pericardial rub. Radiography showed a large shadow in the cardiac region, presumably due to a pericardial effusion. The author has since observed the same sign of absence of respiratory abdominal movement in a dozen cases of rheumatic pericarditis. It is generally more marked in dry pericarditis than in pericarditis with effusion.

### Urobilin in Scarlet Fever.

In a contribution to the *Münchener Medizinische Wochenschrift*, Dr. Hildebrandt draws attention to the greatly increased excretion of urobilin in the urine in the great majority of cases of scarlet fever. This urobilinuria follows the same course as the fever and terminates with the fall of temperature, and the intensity of urobilinuria depends to a large extent upon the degree of fever. There is no abnormal excretion of urobilin during convalescence. The author is of opinion that the presence of urobilin in excess in the urine should prove a useful sign in the differential diagnosis of scarlet fever. At the commencement of a case, where the diagnosis is doubtful between scarlet fever, measles, diphtheria, or a simple tonsilitis or pharyngitis, an exaggerated excretion of urobilin favours scarlet fever. According to the author, this unusual excretion of urobilin points to the presence of a concomitant parenchymatous hepatitis, and he suggests that a scarlatinal hepatitis may play as important a rôle in the pathology of cirrhosis of the liver as scarlatinal nephritis in chronic Bright's disease.

### Rectal or Oral Alimentation.

In a very well written leading article, the *Medical Review* discusses at length the whole question of rectal feeding. After dimisssing suppositories as useless, the author quotes the experiments of numerous investigators to show that if any protein food is absorbed *per rectum* at all, the amount is quite insignificant and more than counterbalanced by disadvantages. Saline injections are valuable in certain ways, and the addition of 5 per cent. dextrose is probably useful, since enough of it may be absorbed to check the onset of acidosis. But even in this strength dextrose may cause pain by the osmotic currents set up, and in higher concentrations this is almost certain to occur. When one doubts the efficacy of rectal feeding, its drawbacks become very prominent. The nursing difficulties are obvious, but in addition it is established that nutrient enemata provoke the secretion of gastric juice. It does not secure physiological rest to the stomach, the author points out, to allow this juice to be poured over an ulcerated surface without having any food on which to act. To neutralise the juice, the patient should have a bismuth lozenge to suck. Ice and preparations of glycerine are both condemned for this usage, but plain hot water, to which a little permanganate of potash has been added, is commended. In the Lenhartz method of immediate feeding with small and frequently repeated quantities of iced milk and beaten-up egg, we have a safer, simpler, and more effective treatment for gastric ulcer, free from disagreeable features. If for any reason this is considered inadvisable, rectal salines with 5 per cent. of dextrose may be used for a time. Between these two procedures it is doubtful if there is any place left to-day in therapeutics for the nutrient enema containing protein or fat.

### Intestinal Antisepsis.

The possibilities of attaining a fair degree of intestinal antisepsis are discussed by Dr. H. C. Wood in the *Therapeutic Gazette*. As a result of collating the researches of several observers, he has come to the conclusion that it is quite possible enormously to diminish the bacterial flora of the intestinal tract, and in fact to reduce them to 1 per cent. or less of their previous abundance. He then proceeds to inquire what drug offers the greatest probability of being useful therapeutically for this purpose. A table is constructed showing in order of merit the drugs which experience has shown to be most effective; all these are pharmacopœial; proprietary drugs and preparations are excluded. Three substances stand out strikingly as being efficient in doses which are within the limits of safety. These are beta-naphthol, formaldehyde, and creosote. Formaldehyde is believed to be absorbed so rapidly that as a practical intestinal antiseptic it is not of much value. Creosote is also, contrary to the commonly accepted opinion, absorbed with a fair degree of rapidity, according to this author. For disinfecting the duodenum he thinks it may be a suitable drug, but not for situations more remote from the mouth. Beta-naphthol, however, is soluble only 1 in 950 at 77° F. in water; though it is probably rather more soluble than this in the bowel contents. Still, in any case, it is relatively insoluble, and has the further advantage of having a good antiseptic action even in very dilute solution. It is therefore the remedy of choice in all cases where it is desired to influnce bacteria in any part of the intestinal tract below the duodenum.

### Undiluted Citrate Milk.

In the *Guy's Hospital Gazette* Dr. H. C. Mann explains very clearly the method of feeding infants on undiluted cow's milk which has been for three years adopted at the Evelina Hospital. So great a revolution is bound to excite incredulity, but the author acknowledges himself a converted sceptic, and his list of cases showing the weekly weight of the infants concerned is one which speaks for itself. Only six babies out of his last four hundred have had to be placed upon other forms of diet. Three facts, he says, have to be remembered: that the great stumbling-block to digestion is the casein clot from cow's milk, and that mere dilution does not remove this difficulty; secondly, that the addition of sodium citrate to each undiluted feed leads to the formation of a much less dense clot; lastly, that the bulk of each feed is small, and hence vomiting is much less likely. In practice the mother is provided with a solution of sodium citrate in water containing as many grains to the drachm as there are ounces in each feed. She is told to put a teaspoonful into the milk, to stir and warm until the food is steaming, and then to cool down to the proper temperature. The amount of each feed and the total quantity per diem depend upon the size of the child and not upon its age. At six months and upwards two pints in twenty-four hours can be taken by many children; but even at a month and less still there is ample evidence in Dr. Mann's statistics to show that small infants can thrive remarkably well on this undiluted citrated cow's milk.

### Submucous Resection of the Septum Nasi.

Although the operation of submucous resection is a comparatively recent one, its popularity is such that it has distanced all competitors. In the *Johns Hopkins Hospital Bulletin*, Dr. Rosenheim summarises his experience of its merits and its limitations. It is a method by which all varieties of deflected septum can be corrected; but it is necessary to be cautious in the case of young children, and to do the operation only when it is urgently demanded. Chronic sinus infections are not a contraindication; in fact, the operation is often a necessary accompaniment of sinus work in order to secure free drainage. Such operations, as well as inferior or middle turbinectomies, are best done at the same time. Luetic individuals who have been well treated and show no signs of the disease yield as satisfactory results as others. It is absolutely essential to have strict asepsis in all details of the technique; infection following the operation should be as infrequent as after any other operation aseptically carried out. Perforations of the septum may be caused infrequently; but they are of no consequence and cause the patient no discomfort. There is no reason to fear sinking of the bridge of the nose following at any time after the operation; on the contrary, external deformities which are present are frequently relieved.

### Arsenic in the Culture of Vines.

M. Schloesing, Junr., read a paper before the Académie des Sciences de Paris with reference to recent work of MM. Moreau and Tinet on the action of lead arseniate in viticulture. The authors, while maintaining that nicotine was the best insecticide to employ, have proved that lead arseniate does not remain as such in the vines and consequently does not seem to be dangerous to use. The President, M. Armand Gautier, in criticising the report, stated that the question of using arsenical preparations in the growing of vines was one which had been carefully studied by the Academy of Medicine, and that that body had come to the conclusion that such a practice should be condemned.

### Fœtid Breath.

An interesting contribution by Dr. Lautmann appears in the *Journal de Médecine de Paris*, on fœtid breath. The various recognised conditions giving rise to fœtid breath, rhinitis, inflammations of the sinuses, bronchiectasis, cancer of the tonsils, sequestræ in the nose, bad teeth, are enumerated, but the author points out that in the great majority of these cases other symptoms and signs present themselves, of which the patient himself complains and for which he seeks medical assistance, so that the diagnosis presents no difficulties. In those cases, however, in which fœtid breath is the sole symptom, the cause is not so obvious and is often difficult to find. In many of these cases the author has been able to discover the origin of the trouble in the fœtid caseous concretions in the tonsils. He cites several cases in which these concretions have been by no means easy to find. The tonsils may be entirely hidden from view by the pillars of the fauces, and the fœtid concretion may lie in the subtonsillar fossa. Simple inspection may be entirely negative, but a jet of fluid directed into the fossa from a syringe forces out the concretion and usually entirely removes the trouble.

### The Ultimate Results of Breast-Feeding.

Dr. Comby, in the *Archives de Méde. des Enjanis*, analyses a thesis of Dr. Roger Simon on the late effects of methods of infant feeding. The latter writer concludes (1) that bottle feeding does not give such good results as breast feeding, (2) that bottle feeding in the home is better than the same carried out a distance from the family circle, (3) that the longer breast feeding is carried out the better is the ultimate result. It seems that the mode of feeding influences the height of the subject long after it has been discontinued, but Dr. Simon considers that breast feeding no longer influences height when continued longer than ten to thirteen months. Dr. Comby states, however, that there are many cases on record where breast feeding has been continued for eighteen to twenty months, or even for years, without affecting the health of the child. He nevertheless insists that after ten months or so, breast feeding should be supplemented by other food, such as soups, broths, etc. Dr. Simon concludes from his researches that women brought up on the breast menstruate earlier than the bottle-fed, and that among the former there are less who eventually suffer from gastro-intestinal troubles than among the latter. Dr. Comby thus thinks that the writer of the thesis is justified in affirming that the superiority of natural over artificial feeding is proved not only in the infant, but in the adult also. The work had been carried out at the Baudelocque clinic.

### Nasal Neuralgia.

Broeckaert, in the *Annales des Maladies du nez et des oreilles*, describes the technique he employs in cases of that type of nasal neuralgia for the relief of which it is necessary to remove the nasal branches of the ophthalmic nerve. He starts by making an incision from the supra-orbital notch to below the internal angle of the eye, following the internal border of the orbit. The two lips of the incision are freed and elevated and the fibres of the orbicularis divided. Two vessels then come into view, the facial artery internally, and the angular vein externally. These are isolated and the nasal branch of the ophthalmic nerve comes into view. This is torn away sharply and violently from its central end. The fronto-palpebral branches are sought for and treated in similar fashion. The wound is then sutured unless the internal nasal nerves are to be found also. For this the same incision suffices, but the nerve must be laid bare before its entry into the internal and anterior orbital canal. For this all the structures as far as the internal bony border of the orbit must be divided, during which time the pulley of the superior oblique muscle must be held inwards. At a depth of 2 centimetres the nerve can be seen stretched between the orbital canal and the periosteum turned outwards; it is accompanied by the ethmoidal artery, which is easily separated. The nerve is then torn away as before. The author is very insistent on the necessity of the violent tearing away of the central end of the nerve. Simple division is useless.

